# Identification of microsatellite loci in *Pinus tecunumanii*

**DOI:** 10.1186/1753-6561-5-S7-P1

**Published:** 2011-09-13

**Authors:** Valderês Aparecida de Sousa, Camila C Mantello, Ananda Virginia de Aguiar, Daiane Rigoni Kresting, Anete Pereira de Souza, Laila Toniol Cardin

**Affiliations:** 1 Embrapa Forestry, Brazilian Agricultural Research Corporation, Colombo, Paraná, 83411-000, Brazil; 2 UNICAMP, Campinas, São Paulo, 13083-970, Brazil; 3 UNESP, Ilha Solteira, São Pauloa, 15385-000, Brazil

## Background

*Pinus tecunumanii* has displayed good performance in tropical regions of Brazil and showed high potential for commercial exploitation. Embrapa Forestry and its partners own many of the species seed production areas. In spite of its importance, the majority of P. tecunumanii germplasm collections remain still genetically uncharacterized. Thus identifying genetic markers is an important tool to genetically characterize these collections.We describe the initial steps to develop microsatellites for *Pinus tecunumanii* by enriched library construction with the ultimate goal of characterizing accessions of the germplasm collections of EMBRAPA.

## Methods

The genomic-enriched library was constructed following the protocol described by [1]. The genomic DNA of *P. tecunumanii* was digested with AFA*I* and enriched in (CT)_8_ and (GT)_8_ repeats. Enriched fragments were amplified by polymerase chain reaction (PCR), connected to a pGEM T-easy vector and transformed into competent XL1- blue *Escherichia coli* cells. The positive clones were selected using the B-galactosidase gene and then grown overnight in an HM/F medium with ampicillin. After PCR 95 positive clones were sequenced in both directions using the T7 and SP6 primers as well as the Big Dye terminator Kit. The sequences were assembled and edited in Seqman (DNAStar), the repetitive regions were found using the Simple Sequence Repeat Identification Tool [2]. Primer select (DNAStar) and Primers Plus were used to design primer pairs flanking the microsatellite regions.

## Results and conclusion

Of the ninety five sequences cloned only eleven contained microsatellite sequences and five showed repeats and adequate flanking regions for primer design. The observed proportion of dinucleotide was 5.26% (5), while trinucleotide and tetranucleotide proportions were 1.05% (1) and 5.26% (5), respectively. Ninety one percent of nucleotides were simply perfect and 9% were compost perfect. The explanation for this low yield (11.6%) can be attributed to the genomic-enriched procedure. To overcome this problem this procedure will be repeated. The obtained sequences will be used for validation of *P. tecunumanii* microsatellite primers and used to estimate the genetic diversity from the germplasm collection located in various regions of Brazil.
